# Seasonal responses and host uniqueness of gut microbiome of Japanese macaques in lowland Yakushima

**DOI:** 10.1186/s42523-022-00205-9

**Published:** 2022-09-27

**Authors:** Akiko Sawada, Takashi Hayakawa, Yosuke Kurihara, Wanyi Lee, Goro Hanya

**Affiliations:** 1 Primate Research Institute, Kyoto University, Inuyama, Aichi 484-8506 Japan; 2 Academy of Emerging Sciences, Chubu University, Kasugai, Aichi 487-8501 Japan; 3 Wildlife Research Center, Kyoto University, Kyoto, 606-8203 Japan; 4 Faculty of Environmental Earth Science, Hokkaido University, Sapporo, Hokkaido 060-0810 Japan; 5 Japan Monkey Centre, Inuyama, Aichi 484-0081 Japan; 6 Center for Education and Research in Field Sciences, Faculty of Agriculture, Shizuoka University, Hamamatsu, Shizuoka, 431-3532 Japan; 7 Center for Ecological Research, Kyoto University, Inuyama Campus, Inuyama, Aichi 484-8506 Japan

**Keywords:** Wild animal, Nonhuman primates, Food intake, Diet shift, Gut bacteria, 16S rRNA

## Abstract

**Background:**

Changes in the gut microbial composition is an important response to cope with the seasonal fluctuations in the environment such as food availability. We examined the bacterial gut microbiome of the wild nonhuman primate, Japanese macaque (*Macaca fuscata*) in Yakushima over 13 months by noninvasive continuous sampling from three identified adult females.

**Results:**

Dietary composition varied considerably over the study period and displayed marked shifts with the seasons. Feeding of leaves, fruits, and invertebrates were their main foods for at least one month. Diet had a significant influence on the gut microbiome. We also confirmed significant effect of host uniqueness in the gut microbiome among the three macaques. Leaf-dominated diet shaped unique gut microbiome structures where the macaques had the highest alpha diversity and their gut microbiome was enriched with Spirochaetes and Tenericutes. Diet-related differences in the putative function were detected, such as a differentially abundant urea cycle during the leaf-feeding season.

**Conclusion:**

Both diet and host individuality exerted similar amounts of effect on gut microbe community composition. Major bacterial taxa showed a similar response to monthly fluctuations of fruit and invertebrate feeding, which was largely opposite to that of leaf feeding. The main constituents of fruits and invertebrates are both digestible with the enzyme of the host animals, but that of leaves is not available as an energy source without the aid of the fermentation of the gut microbiome.

**Supplementary Information:**

The online version contains supplementary material available at 10.1186/s42523-022-00205-9.

## Introduction

A number of studies have described a complex relationship between hosts and their gut microbe. There is a continuum in the degree of dependence on symbiotic microorganisms by animals, from complete reliance to the lack of beneficial symbionts [1]. For example, in primates, cows, and termites, gut microbes play indispensable roles in the digestion of foods [2–4]. Factors influencing gut microbial structures are host phylogeny, health, social groups as well as diet and habitat [5, 6]. Above all, gut microbiota is influenced by the host’s diet in many mammals [7, 8]. Along with behavioral changes [9, 10], altering the gut microbiome may be one possible physiological adaptation to cope with seasonal fluctuations of food availability by modifying digestion and fat storage [11–13]. For example, in black howler monkeys, the abundance of Ruminococcaceae, active cellulose degraders, was the highest during periods of low quality food [14].

Host uniqueness is one of the important yet unexplored parameters in the studies of the gut microbiome of wild animals. The gut microbiome is host-individual specific, settled throughout the individual’s lifetime [15], and it is modified via the sociality of the individual’s conspecifics [16, 17]. Different human individuals retain a high host uniqueness of the gut microbiome, despite the intervention of dietary fiber intake [18]. If host uniqueness is a significant factor in shaping the gut microbiome, the seasonal pattern based on a small number of samples per focal individual may be confounded by host uniqueness. It would be difficult to distinguish resident and transient microorganisms by such cross-sectional sampling of many individuals, which may cause overestimation of the roles of the species that appear only temporarily in the hosts’ gut [1]. A large number of samples per individual covering multiple seasons is necessary to separate the effects of host uniqueness and seasonality. Such data for wild animals has been limited due to the difficulties in collecting samples from the identical individual repeatedly over a sufficiently long period.

Seasonality, or the dietary shift in general, and host uniqueness in the gut microbiota may be linked to each other. There is often a redundancy in the function of bacteria [19]; therefore, a different set of gut microbes among different individuals may have a similar function. However, if particular bacterial taxa having a unique function is necessary to digest a particular food for that season, gut microbiome among different individuals may converge in that season. One such notable example is the change in gut microbe of infant mammals. Among human infants, convergence of the gut microbiota accelerates at 2–4 months of age, but individuality increases again when the infant starts eating solid foods [20]. The accelerated convergence was due to a bloom of *Bifidobacterium*, a genus capable of metabolizing oligosaccharides, thus causing adaptive response to a special diet (milk) before weaning.

Japanese macaques (*Macaca fuscata*) are the endemic and only nonhuman primate species in the Japanese archipelago. Unlike other nonhuman primates living in the tropics, their habitats are cold- and warm-temperate forests where the macaques experience cold winter as well as large seasonal fluctuations in food availability [21]. For example, even though some foods (e.g. mature leaves) are available year-round, most high-quality foods, such as fruits, seeds and young leaves, are available only during a limited periods of the year [21, 22]. Wild Japanese macaques in Yakushima (*M. f. yakui*), the southernmost population, are dietary generalists in which no single food type (fruit, seed, mature leaf, young leaf, flower, bark, fungus, invertebrate, etc.) dominates their diet on an annual basis and dietary composition changes seasonally [23]. Such dietary shifts may lead to dynamic seasonal changes in the gut microbiome in Yakushima Japanese macaques. The wild macaques in the western lowland forest of Yakushima Island, investigated in this study, were well habituated to the presence of researchers, which allowed close observation and repeated fecal sampling from identical individuals.

The aim of this study was to reveal the effects of seasonal dietary fluctuations and host individuality on the gut bacterial microbiome of three individuals of wild Japanese macaques. First, we compared the amplitudes of the effects of two factors on the alpha and beta diversity indices of the gut microbiome. Second, we investigated the seasonal changes in the abundance of bacterial taxa and their putative function to examine the hypothesis that a host possesses gut microbiota capable of digesting the host’s foods in that season. Specifically, we predict whether fibrolytic taxa or fiber-digesting function increases during leaf-feeding seasons. For that purpose, we examine the correlations between the abundance of major bacterial taxa and the intake of major food categories. Furthermore, Linear discriminant analysis Effect Size (LEfSe) is used to detect differentially abundant taxa and putative function in different seasons.

## Materials and methods

### Study site

The study site was in the western lowland forest of Yakushima Island, Japan (30°N, 130°E). Primary and secondary warm temperate evergreen broad-leaved forest covered the study area [24]. The mean annual temperature during the study period was 18.9 °C and mean annual rainfall was 2659.8 mm [25].

### Behavioral observation and fecal sample collection

We conducted behavioral observation and fresh fecal sample collection from three identified adult female Japanese macaques in two neighboring groups (“Em” from the KwCE group; “Bo” and “Fl” from the KwA group) from October 2012 to October 2013 (13 months). We changed the focal groups at least every three days and tried to distribute observation days as evenly as possible within each month. The total contact time of these two groups was 574 h (44.2 ± 30.5 h per focal animal; 44.2 ± 13.6 h per month), and the observation time for the three focal females was 234 h. All members of the KwCE and KwA groups were well-habituated to the presence of observers and had never been under the influence of provisioning food. One of the authors (YK) conducted 1-h focal animal sampling to collect behavioral data, which were presented as a part of previously published works [26, 27]. The onset and the end of feeding measured to the nearest second and food species and part eaten were recorded. We also recorded the feeding rate (#food units/sec) for each food as long as possible.

We collected a total of 85 feces (29 for Bo, 28 for Em, and 28 for Fl). Because we conducted behavioral observation and fecal sample collection at the same time, it was difficult to collect samples in a predetermined cycle. We provide exact dates of sample collection in Additional file 1. We placed all feces in clean (non-contaminated by biological materials) plastic bags immediately after defecation, and then stored them in a thermos bottle containing frozen refrigerants. We brought these samples back to the field station, which was located approximately 6 km away from the field site, within 10 h from collection. We temporarily stored the samples in a − 30 °C freezer and subsequently transferred them to our laboratory using a cold chain. We stored the samples at − 30 °C until analysis.

### DNA extraction, purification, library preparation, and amplicon sequencing

We followed laboratory procedures of Hayakawa et al. [28] for microbial DNA analysis. Prior to DNA extraction by QIAamp DNA Stool Mini Kit (®QIAGEN GmbH), we cut the frozen fecal samples into two pieces on dry ice to avoid thawing. Then, to avoid possible soil-borne contamination, we shaved off the inner part of the fecal samples from the cross-sectional surface and stored it in a 2-ml plastic tube. After freeze-drying to decrease the effect of freeze-thawing, we mixed each sample with 1.4 ml of Buffer ASL supplied by the kit. We crushed the mixture with four zirconia beads (3 mm in diameter) and 1 mg of zirconia/silica beads (0.1 mm in diameter) at 4200 rpm for 4 min or more. We extracted and purified total fecal DNA according to the manufacturer’s protocol. We eluted the purified DNA in 100 μl of Buffer AE with 30 min of incubation at ambient temperature on the column. We quantified the DNA concentration with a Qubit dsDNA HS Assay Kit (®Thermo Fisher Scientific).

We performed amplicon library preparation for the MiSeq platform (®Illumina) according to the manufacturer’s protocol (Part # 15044223 Rev. A) but with a modified duration of incubation of 70 °C (1 h) [28]. We amplified the V1–V2 region of the bacterial 16S ribosomal RNA gene by PCR. We used 27Fmod and 338R primers [29] fused with the specific overhang adapters as the forward and reverse primers, respectively (5´-TCG TCG GCA GCG TCA GAT GTG TAT AAG AGA CAG—[forward primer overhang adapter]—AGR GTT TGA TYM TGG CTC AG—[27Fmod]-3´and 5´-GTC TCG TGG GCT CGG AGA TGT GTA TAA GAG ACA G—[reverse primer overhang adapter]—TGC TGC CTC CCG TAG GAG T—[338R]-3´). We performed the PCR using KAPA HiFi HS ReadyMix (® Nippon Genetics) with 10 μM primer each and 12.5 ng DNA as the template in a total volume of 25 μl under the thermal conditions of 95 °C for 3 min as initial denaturation and 18 thermal cycles at 98 °C for 30 s as denaturation, 55 °C for 30 s as primer annealing, and 72 °C for 30 s as an extension, followed by the final extension at 72 °C for 5 min. We purified 20 μl of each PCR product using 36 μl Agencourt AMPure XP (® Beckman Coulter). The purified PCR product was eluted in 42 μl of 10 mM Tris–Cl (pH 8.5). Using KAPA HiFi HS ReadyMix and a Nextera XT Index Kit (® Illumina), we conducted the second PCR to attach the specific dual indices and sequencing adapters in a total volume of 50 μl mixture containing 5 μl forward primer, 5 μl reverse primer, and 5 μl purified first PCR solution with 8 thermal cycles. We purified the second PCR product using Agencourt AMPure XP and eluted it in 27.5 μl of 10 mM Tris–Cl (pH 8.5). We measured the DNA concentration of each purified second PCR product using a Qubit dsDNA HS Assay Kit and estimated the fragment size distribution and molarity using an Agilent 2100 Bioanalyzer and DNA1000 Kit (® Agilent Technologies). The libraries were subjected to a run with other libraries unrelated to this study and 5% PhiX spike-in on an Illumina MiSeq (®Illumina) using MiSeq Regent Kit v3 (600 cycles) under the MiSeq Control Software v2. Read lengths in the MiSeq run were 301 bp (forward sequences), 8 bp (forward indices), 8 bp (reverse indices), and 301 bp (reverse sequences).

### Sequence processing and quality control

In the bioinformatics procedures, we mainly used Claident v0.2.2016.04.07 (https://www.claident.org/) and QIIME2 v2021.11 (http://qiime.org/). We converted the MiSeq base calls to FASTQ files using *configureBclToFastq.pl* implemented by bcl2fastq Conversion Software v1.8.4 with options –no-eamss, –mismatches 0 and –use-bases-mask Y300n,Y8,Y8,Y300n. We demultiplexed the FASTQ files using *clsplitseq* in Claident with the option–minqualtag = 30 to discard read pairs with low quality index sequences, where the index sequences included nucleotide(s) with a < 30 quality score. We performed quality control, denoising, and chimera removal, and generated the amplicon sequence variants (ASVs) using the DADA2 pipeline in QIIME2. Rarefaction curves on the number of detected ASVs showed the enough number of reads sequenced in each sample (Additional file 2). The phylogenetic tree of the ASVs was generated using *qiime phylogeny align-to-tree-mafft-fasttree*. To assign the taxonomy of the ASVs, we used the QIIME2 weighted taxonomic classifier with Greengenes 13_8 reference database [30]. To explore the functional difference, we predicted the Kyoto Encyclopedia of Genes and Genome Orthology (KO) pathways through phylogenetic investigation of communities by reconstruction of unobserved states (PICRUSt2) [31] following the guidelines at https://github.com/picrust/picrust2/wiki.

### Statistical analysis

Unless otherwise indicated, we conducted statistical analyses in R v. 3.6.1 (https://www.r-project.org/). We pooled all observational data to calculate monthly macaque diet composition. This contradicts to the purpose of this study to reveal the relative effects of individuality and seasonality, but food repertoire diversity as well as energy intake did not differ between the two groups [27]. In addition, in this population, the group is cohesive, and thus synchronization of feeding is high within the group [32, 33]. Because the observation time per individual in each month was limited, the dietary record for a particular individual for a particular month is likely to be biased, so pooling data among various individuals is a better way to know the average dietary composition in that month. We categorized food items into four types: fruits/seeds, leaves (including buds and shoots), invertebrates, and the remaining others (e.g., pith, bark, fungi, unidentified items). We evaluated the dietary composition based on the dry weight intake calculated by feeding time, feeding rate, and dry weight of the foods [34]. To categorize the 13 study months into a smaller number of dietary seasons, we conducted clustering analysis by Ward’s hierarchical agglomerative clustering method using *stat* and *cluster* packages.

In the analysis of the gut microbiome, for alpha diversity, we generate observed ASVs, Shannon’s index using function *estimate_richness* embedded in the R package *phyloseq*, and Faith’s PD with the R package *btools*. We used Kruskal Wallis rank sum test and Dunn’s test (*p*-adjustment by Bonferroni) to examine whether alpha diversity indices differed by seasons. For beta diversity, we generated the Bray–Curtis, unweighted and weighted UniFrac distances and examined the effect of diet and host uniqueness in two ways. First, we examined the effects of proportion of dry weight intake of the four food categories (fruit/seed, leaf, insect, and others) in each month and individual identity on diversity indices by multiple regression on distance matrices (MRM) using R package *ecodist*. We calculated the Gower distance for the monthly diet data and the individual from which the samples were collected. Second, we examined whether beta diversity indices differed among the individuals and the dietary seasons (defined by cluster analysis) by PERMANOVA using the *adonis* function of R (permutation = 999). To find the bacterial taxa that changed the abundance in response to the monthly changes in diet, we conducted Spearman’s rank correlation between abundance of the top five taxa at phylum, family, and genus levels and the feeding times of the three major foods (mature + young leaves, fruits + seeds and invertebrates). We adjusted *P* values with the false discovery rate (FDR). To find bacterial taxa and KO pathways enriched in each season (LDA score > 2.0, *P* < 0.05), we conducted LEfSe (http://huttenhower.sph.harvard.edu/galaxy/).

## Results

### Sequencing results

**Fig. 1:**
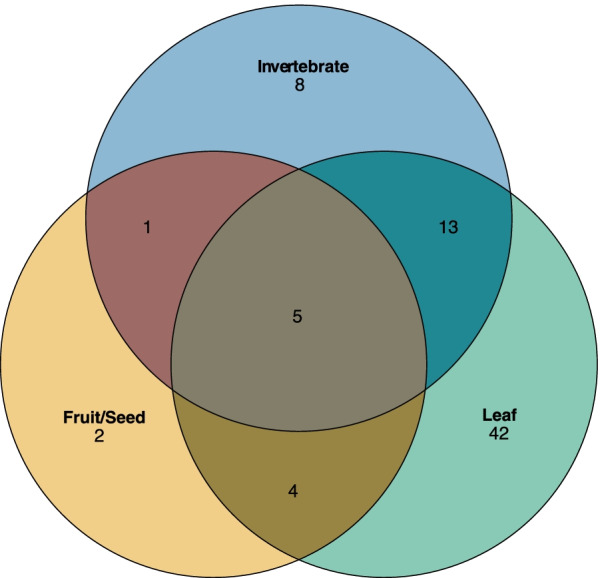
Venn diagram of overlapping core taxa with relative abundance no less than 0.1% and present in 75% of the samples among dietary seasons

After quality filtering, we obtained 10,986,255 reads from a total of 84 fecal samples (one sample did not pass denoising) (Additional file 3). Taxonomic assignment revealed representatives (core taxa) from 9 phyla, 20 classes, 33 orders, 66 families, and 110 genera (Fig. 1). Top-five taxa were Firmicutes (57.3%), Bacteroidetes (33.7%), Spirochaetes (4.8%), Actinobacteria (1.5%), and Tenericutes (0.2%) at the phylum, Lachnospiraceae (31.1%), Ruminococcaceae (29.6%), Prevotellaceae (17.8%), Erysipelotrichaceae (3.7%), and Spirochaetaceae (2.7%) at the family, and, *Prevotella* (13.8%), *Blautia* (5.0%), *Faecalibacterium* (4.4%), *Coprococcus* (3.4%), *Ruminococcus* (2.6%) at the genus levels (Additional file 4).

### Diet composition across seasons

**Fig. 2:**
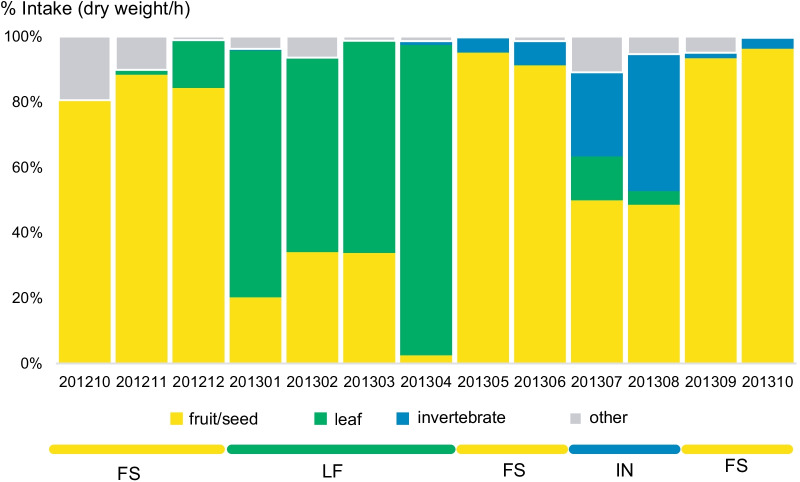
Monthly variations in the diet of Japanese macaques and the classification of the three dietary seasons. *FS* fruit/seed-feeding; *LF* leaf-feeding; *IN* invertebrate-feeding seasons

Japanese macaques displayed marked dietary shifts with the seasons (Fig. 2). We classified the study period into three dietary seasons indicated by cluster analysis (Additional file 5): leaf-feeding (LF: January-April 2013; *N* of fecal samples = 29), invertebrate-feeding (IN: July and August 2013; *N* = 15), and fruit/seed-feeding (FS: other months; *N* = 40) seasons.

### Overall trends of gut microbial diversity

**Fig. 3:**
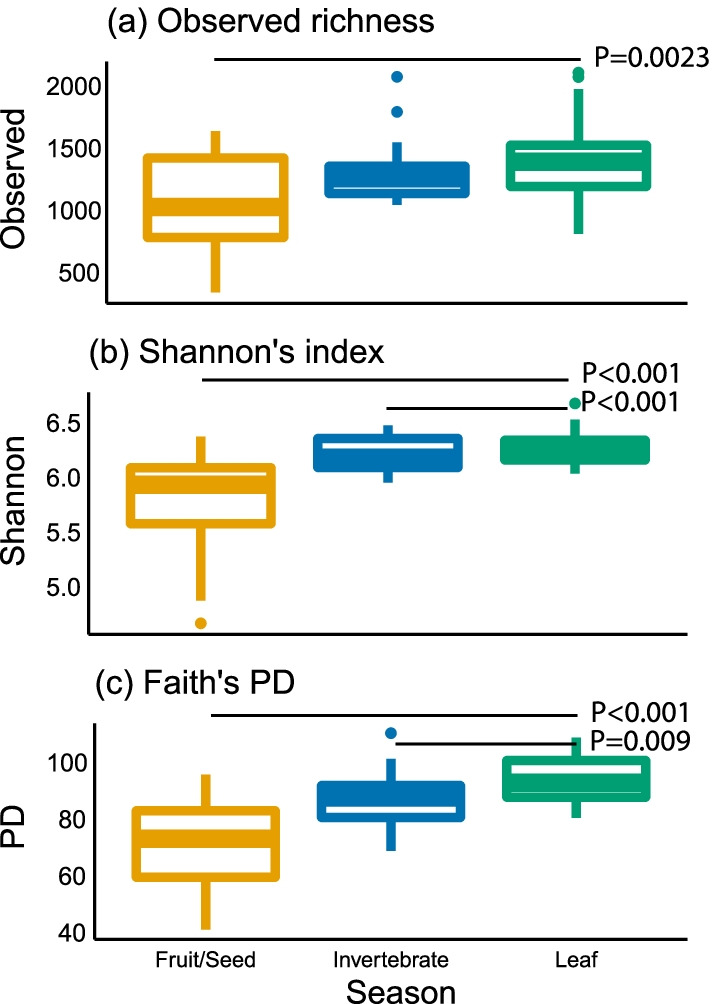
Alpha diversity indices of Japanese macaque gut microbiota. (**a**) Observed richness. (**b**) Shannon’s diversity index. (**c**) Faith’s phylogenetic diversity (PD)

All of the alpha diversity indices were the highest during the LF season (observed richness: Kruskal–Wallis *χ*^2^ = 11.1; Shannon’s diversity index: *χ*^2^ = 32.0; Faith’s PD: *χ*^2^ = 34.8; df = 2, *P* < 0.0001 for all diversity indices; Fig. 3). MRM analysis indicated that the effect of diet composition on alpha diversity indices was significant but that of the host individual was not (Table 1).

**Table 1:** Summary of the results on multiple regression on distance matrices (MRM) on the effect of diet and individuality on gut microbe alpha diversity indices

Diversity index	Factor	Coefficient	*P*
Observed richness	Intercept	1630.423	0.958
(*R*^2^ = 0.007, *P* = 0.014)	Diet	−0.016	0.249
	Individuality	0.081	0.016
Shannon index	Intercept	1589.140	0.975
(*R*^2^ = 0.008, *P* = 0.019)	Diet	−0.001	0.932
	Individuality	0.090	0.018
Faith's PD	Intercept	1294.915	1
(*R*^2^ = 0.047, *P* = 0.001)	Diet	0.043	0.025
	Individuality	0.214	0.001

**Fig. 4:**
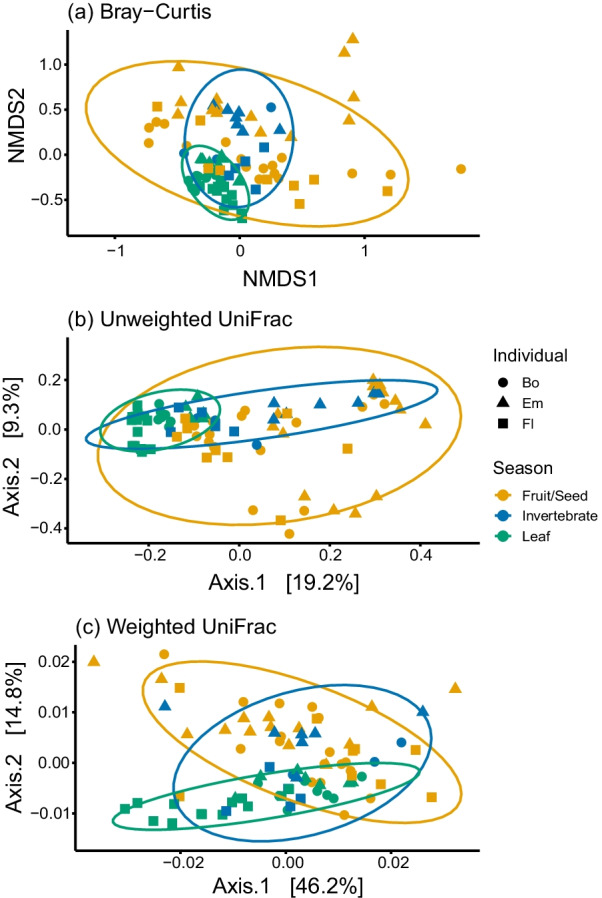
Beta diversity indices of Japanese macaque gut microbiome among different seasons and individuals. (**a**) NMDS (nonmetric multidimensional scaling) by Bray–Curtis dissimilarity; (**b**) PCoA (principal coordinate analysis) by Unweighted UniFrac distance, (**c**) Weighted UniFrac distance

Both the diet composition and host individual significantly affected the beta diversity of the gut microbiota. According to MRM analysis, the relative importance varied among the diversity indices: the effects were similar for weighted UniFrac, and the effect of individuality was larger than that of diet for unweighted UniFrac and Bray–Curtis dissimilarities (Table 2). PERMANOVA analysis also revealed a significant influence of the three dietary seasons (LF vs. FR vs. IN) on gut microbiome (Bray–Curtis: *R*^2^ = 0.10; weighted UniFrac: *R*^2^ = 0.11; unweighted UniFrac: *R*^2^ = 0.13; *P* < 0.0001 for all the measures; Fig. 4). We also found a significant effect of host individual (Bray–Curtis: *R*^2^ = 0.12; weighted UniFrac: *R*^2^ = 0.11; unweighted UniFrac: *R*^2^ = 0.14; *P* < 0.001; for all measures; Fig. 4), which explained a variance equivalent to dietary seasons.

**Table 2:** Summary of the results on multiple regression on distance matrices (MRM) on the effect of diet and host uniqueness on gut microbe community structure

Diversity index	Factor	Coefficient	*P*
NMDS by Bray–curtis	Intercept	978	1
(*R*^2^ = 0.081, *P* = 0.001)	Diet	0.133	0.002
	Host uniqueness	0.306	0.001
PCoA	Intercept	957	1
(unweighted UniFrac)	Diet	0.181	0.002
(*R*^2^ = 0.082, *P* = 0.001)	Host uniqueness	0.270	0.001
PCoA	Intercept	1246	1
(weighted UniFrac)	Diet	0.138	0.002
(*R*^2^ = 0.034, *P* = 0.001)	Host uniqueness	0.147	0.001

### Correlations between intake of major foods and abundance of the major bacterial taxa

Over the course of the 13 months, we found statistically significant correlations between the intake of the three major foods and abundance of the top five bacterial taxa (Table 3). At the phylum level, leaf-feeding was characterized by higher abundances of Spirochaetes and Tenericutes. At the family level, leaf-feeding was associated with abundances of Spirochaetaceae (+). At the genus level, *Ruminococcus* increased in response to leaf-feeding. Fruit/seed-feeding showed the opposite pattern to leaf-feeding with respect to the abundances of Spirochaetes (−), Tenericutes (−), Spirochaetaceae (−), and *Ruminococcus* (−). Invertebrate-feeding also showed almost the opposite pattern to leaf-feeding, such as Spirochaetes (−), Tenericutes (−), Spirochaetaceae (−), and *Ruminococcus* (−).

**Table 3:** Summary of the Spearman's rank correlations between proportion of food intake of the three major foods and abundance of the top five taxa

		Leaf		Fruit/Seed		Invertebrate	
Taxnomic level	Taxa	Spearman's correlation coefficient (*ρ*)	*P*	Spearman's correlation coefficient (*ρ*)	*P*	Spearman's correlation coefficient (ρ)	*P*
Phylum	Firmicutes	0.059	0.59	−0.081	0.47	0.087	0.43
	Bacteroidetes	0.182	0.10	−0.165	0.13	−0.061	0.58
	Actinobacteria	0.099	0.37	−0.199	0.07	−0.271	0.0125*
	Spirochaetes	0.527	< 0.0001*	−0.646	< 0.0001*	−0.433	< 0.0001*
	Tenericutes	0.606	< 0.0001*	−0.683	< 0.0001*	−0.297	0.0060*
Family	Lachnospiraceae	−0.060	0.59	0.067	0.55	0.213	0.05
	Ruminococcaceae	0.141	0.20	−0.195	0.08	−0.030	0.79
	Prevotellaceae	0.160	0.15	−0.156	0.16	−0.128	0.24
	Erysipelotrichaceae	−0.131	0.24	0.167	0.13	0.305	0.0047*
	Spirochaetaceae	0.523	< 0.0001*	−0.645	< 0.0001*	−0.437	< 0.0001*
Genus	Prevotella	0.160	0.15	−0.156	0.16	−0.128	0.24
	Blautia	−0.206	0.06	0.214	0.05	0.260	0.0171*
	Faecalibacterium	−0.078	0.48	0.030	0.79	0.026	0.82
	Coprococcus	0.010	0.93	−0.019	0.86	−0.091	0.41
	Ruminococcus	0.277	0.0107*	−0.371	0.0005*	−0.346	0.0012*

*Significant after false discovery rate (FDR) correction

### Differentially abundant bacterial taxa and pathways in each dietary season

**Fig. 5:**
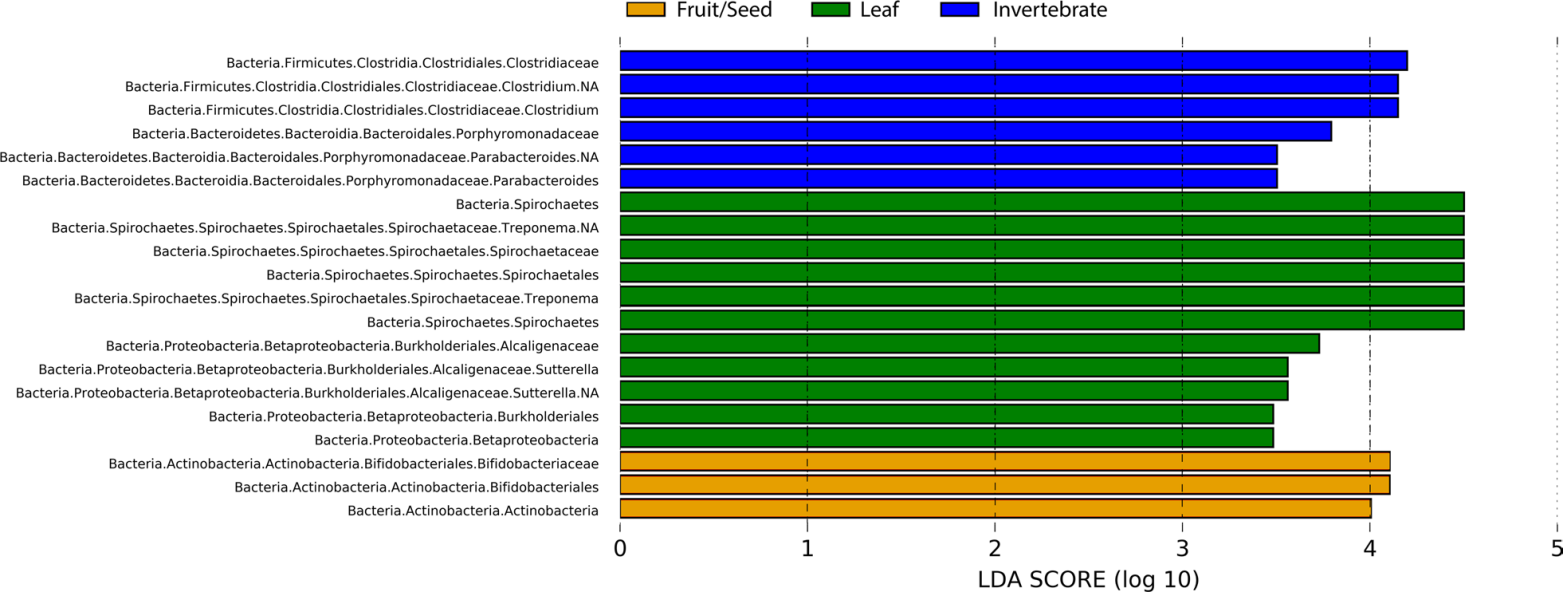
Differentially abundant bacterial taxa among the three dietary seasons estimated by LEfSe

LEfSe analysis (Fig. 5) indicated that the LF season was characterized by the abundance of ASVs of the phyla Spirochaetes and Proteobactreria, such as Spirochaetacea and Alcaligenaceae. In the FS season, ASVs belonging to phylum Actinobacteria, such as Bifidobacteriaceae, were differentially abundant. In the IN season, two families, Clostridiaceae (phylum Firmicutes) and Porphyromonadaceae (phylum Bacteroidetes), were also differentially abundant.

**Fig. 6:**
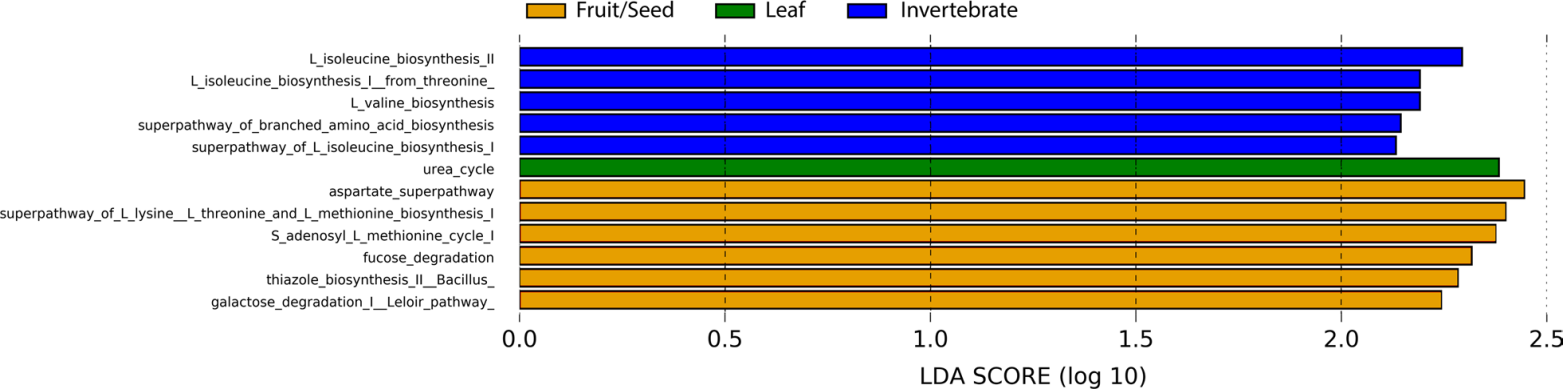
Differentially abundant putative functions of the gut microbiota among the three dietary seasons estimated by LEfSe

By the functional prediction through PICRUSt2 and LEfSe analysis (Fig. 6), the LF season was more enriched with a urea cycle. In the IN season, the pathways of the biosynthesis of two essential amino acids (isoleucine and valine) were differentially abundant. In the FS season, various pathways were differentially abundant, such as those of the synthesis of several amino acids (e.g. aspartate, l-lysin, threonine, methionine) and degradation of simple sugars (fucose and galactose).

## Discussion

### Host uniqueness

We detected a statistically significant effect of host uniqueness. A study on Japanese people showed that intra-individual variability was consistently smaller than inter-individual variability in spite of dietary fluctuations [35], which is consistent with our finding on wild Japanese macaques. It is difficult to compare dietary variability directly between modern humans and wild animals, but there is often virtually no overlap in dietary composition among different months in our study subjects, and this is common for many other wild nonhuman primates [36], which are largely dietary generalists [37]. Even though we have to be cautious about generalizing this finding, since it is based on only three individuals, it is surprising that wild Japanese macaques possessed an individuality in the gut microbe whose effect was comparable with that of seasonal variations in the diet. Therefore, more studies are required to further explore the relative explanatory power of individuality compared with other factors. Primatologists have repeatedly shown that diet has a significant influence on the gut microbiota, but this study implies that it is necessary to consider whether a hidden effect of individuality may have contributed to an overestimation of the effect of diet. For example, the composition of gut microbiota was different between the two sub-populations within Yakushima having a contrasting diet [12]. The NMDS and other beta diversity plots of that study indicated that there is no overlap between the two sub-populations, which stands in contrast to the results of this study (Fig. 4), showing considerable overlap among dietary seasons. In the study on the two subpopulations in Yakushima, the fact that the samples were from different individuals may have exaggerated the effect of diet. One solution is to sample repeatedly from multiple same individuals and control the effect of individuality statistically, for example by generalized linear mixed model. If this is impossible, we recommend sampling as many individuals as possible [38].

### Seasonal changes in the composition of gut microbiota

Japanese macaques exhibited high microbial plasticity in response to different diets. Such strong association has also been reported in many other nonhuman primates [14, 39]. Our data further strengthened the evidence for a widespread tendency across the entire order. However, the responses of the respective taxa may not be similar to those reported for other species of animals.

Leaf-feeding was characterized with high abundances of Tenericutes and Spirochaetes. In American bisons (*Bison bison*), Tenericutes increased with consumption of high-protein plants [40]. We can regard this as a similar finding with a positive correlation in Japanese macaques because leaves are typically higher in protein among various plant food items [41]. However, the higher abundance of Spirochaetes in the leaf-feeding season in this study was inconsistent with some of the previous studies. For example, wild gorillas (*Gorilla* spp.) showed decreased Spirochaetes in leaf-feeding season [42]. Wild rhesus macaques (*Macaca mulatta*) also had decreased Spirochaetes in the dry season, when their diet is largely folivorous [43]. Because these variations occur even within the same host genus (*Macaca*), the host’s trait may not be the only reason. One possibility for these inconsistent responses to the diet is the analyzed taxonomic level. A single phylum may contain functionally various species of bacteria that show different responses to the same diet. In fact, at the genus level, the abundances of *Ruminococcus*, which increased during leaf-feeding seasons in this study (Table 2), were also higher in leaf-feeding situations in other primates [43–45]. Another possibility is the methodological differences among studies, such as storage method, primer choice, and bioinformatics processes, which are all known to have an effect [28]. However, regardless of the methods used, relative abundance among samples can be evaluated by any method [28], so it is difficult to assume that these methodological differences generate seemingly different responses.

Another characteristic of the leaf-feeding season was the highest gut microbial diversity. Similar tendencies were reported for Tibetan macaques and humans having a higher gut microbial diversity in leaf-eating winter [46, 47] or with higher intake of high-fiber foods [48]. A study on three lemur species also showed folivorous sifakas (*Propithecus coquereli*) had a greater gut microbial diversity than frugivorous lemurs [49]. These studies may suggest that high alpha diversity enhances the microbial breakdown of polysaccharides, which is a necessary step for animals to use them as an energy source. However, before concluding as such, we need to pay attention to a study indicating inconsistent responses in alpha diversity to a dietary shift even within the same species [50]. In fact, in the highland Yakushima, where macaques are more folivorous, alpha diversity did not differ from that in the lowland where macaques are more frugivorous [12].

An analysis of the relationship between the intake of major foods and the abundance of major bacterial taxa indicated that Japanese macaques showed similar responses to fruit- and invertebrate-feeding (Table 3). Many studies on wild nonhuman primates have reported a marked shift in gut microbiome in response to dietary shift from non-fibrous to fibrous foods, such as from fruits to leaves [14, 42, 46]. The lack of a clear difference between fruit- and invertebrate-feeding in Japanese macaques stands in contrast to these leaf-related dietary shifts. One population of wild capuchin monkeys exhibited responses in gut microbiota to seasonality in the consumption of insects and fruits [51], but another populaiton did not [52]. The main constituents of fruits and invertebrates, such as starch, simple sugar and protein, are digestible with the enzymes of the host animals [52]. However, leaves contain a large amount of cellulose, which is indigestible without the aid of the gut microbe [12]. Because macaques possess chitinase of their own [53], they do not need to depend on gut microbe to digest chitins contained in the exoskeletons of invertebrates. Therefore, the major bacterial taxa exhibit a suite of responses to either leaf- or non-leaf-feeding, which do and do not accompany extensive fiber-fermentation abilities, respectively.

In spite of the general similarity in the response to fruit/seed- and invertebrate-feeding, there have also been different responses. However, again, the response was inconsistent with some of the other animals. For example, even though Bifidobacteriaceae was differentially abundant in the FS season in this study, that family increased with meat-eating among dogs [54]. Clostridiaceae was differentially abundant in the IN season in this study, but that family was more abundant in frugivorous gorilla populations than in folivorous ones [42]. We detected an increase in Erysipelotrichaceae with increasing invertebrate-feeding, and this family also increased when dogs ate meat [54]. However, carp showed a decrease of this family when their diet switched from grass to meat [55].

### Seasonal variations in the putative functions of the gut microbiota

We found differences in the putative functions of the gut microbiota among the three dietary seasons. In the LF season, a pathway of the urea cycle was differentially abundant. When primates obtain energy mostly from leaves, nitrogen intake may become excessive [56], so urea excretion is expected to increase in the kidneys of the host. It has been experimentally confirmed in cows that increased nitrogen intake increased urea concentration in the gut microbiota [57]. The observed increase of the urea cycle in this study is also likely to be a response to a nitrogen-rich folivorous diet. Increased pathways of the degradation of simple sugars (fucose and galactose) in the FS season may be related to the increased intake of simple sugars by fruit-feeding. Interestingly, in a study conducting a metagenomics analysis of Japanese macaques in Yakushima, more frugivorous lowland samples were more enriched with glycogen biosynthesis and d-galacturonate degradation, both of which are related to increased simple sugar intake [12]. However, a detailed examination is difficult because the sugar composition of food fruits for wild primates remains largely unknown. We also found enrichment of biosynthesis of two amino acids (isoleucine and valine) in the IN season, but an interpretation is also difficult here because the amino acid composition of invertebrates and other foods has not been reported for most wild primates.

Unlike our prediction, we did not find higher enrichment with regard to fiber-digesting ability during the LF season. A metagenomic study of the gut microbiota of Japanese macaques revealed that the genes concerning the digestion of fiber, such as cellulose, were uniformly available in both more folivorous highland and frugivorous lowland samples [12]. That study also shows higher fermentation ability in the highland by in vitro fermentation assay. Therefore, even though we did not detect enrichment of fiber-digesting pathways, this does not mean that fiber-digesting ability is similar between LF and other seasons. Fiber-digesting ability may be regulated at a different level, for example, gene expression.

## Conclusion

Both diet and host individual affected the composition of the gut microbiota of wild Japanese macaques. A leaf-based diet shaped unique gut microbial patterns, where the gut microbial diversity became the highest when some bacterial taxa increased the abundance. The major bacterial taxa showed more or less similar responses to the monthly fluctuations in fruit- and invertebrate-feeding time.

## Supplementary Information

**Additional file 1**: Data on sampling days and ID of the fecal samples used in the study and the feeding times (sec) of three focal Japanese macaques during direct observations.

**Additional file 2**: Rarefaction curves on the number of detected ASVs with the increasing number of reads.

**Additional file 3**: Number of reads of ASVs detected in the fecal samples of three individuals of Japanese macaques.

**Additional file 4**: Phylum, family and genus composition of the gut microbiome of Japanese macaques in the three dietary seasons.

**Additional file 5**: Results of cluster analysis on the monthly variations of dietary composition of three focal female Japanese macaques.

## Data Availability

We have deposited the sample metadata and sequences in the DDBJ (DNA Data Bank of Japan) database with accession number PRJDB6774.

## Abbreviations

ASV: Amplicon sequence variant
FDR: False discovery rate
FS: Fruit/seed-feeding
IN: Invertebrate-feeding
KO: Kyoto encyclopedia of genes and genome orthology
LF: Leaf-feeding
LEfSe: Linear discriminant analysis effect size
MRM: Multiple regression on distance matrices
NMDS: Nonmetric multidimensional scaling
PCoA: Principal coordinate analysis
PD: Phylogenetic diversity
PICRUSt2: Phylogenetic investigation of communities by reconstruction of unobserved states
