# Supplementary material for: Seasonal responses and host uniqueness of gut microbiome of Japanese macaques in lowland Yakushima
Source: Anim Microbiome. 2022 Sep 27;4:54. doi: 10.1186/s42523-022-00205-9 (PMC9513907; doi:10.1186/s42523-022-00205-9)

Supplementary Information 1 Rarefaction curves on the number of detected ASVs with the increasing number of reads

Akiko Sawada, Takashi Hayakawa, Yosuke Kurihara, Wanyi Lee, Goro Hanya

Seasonal responses and host uniqueness of gut microbiome of Japanese macaques in the lowland Yakushima

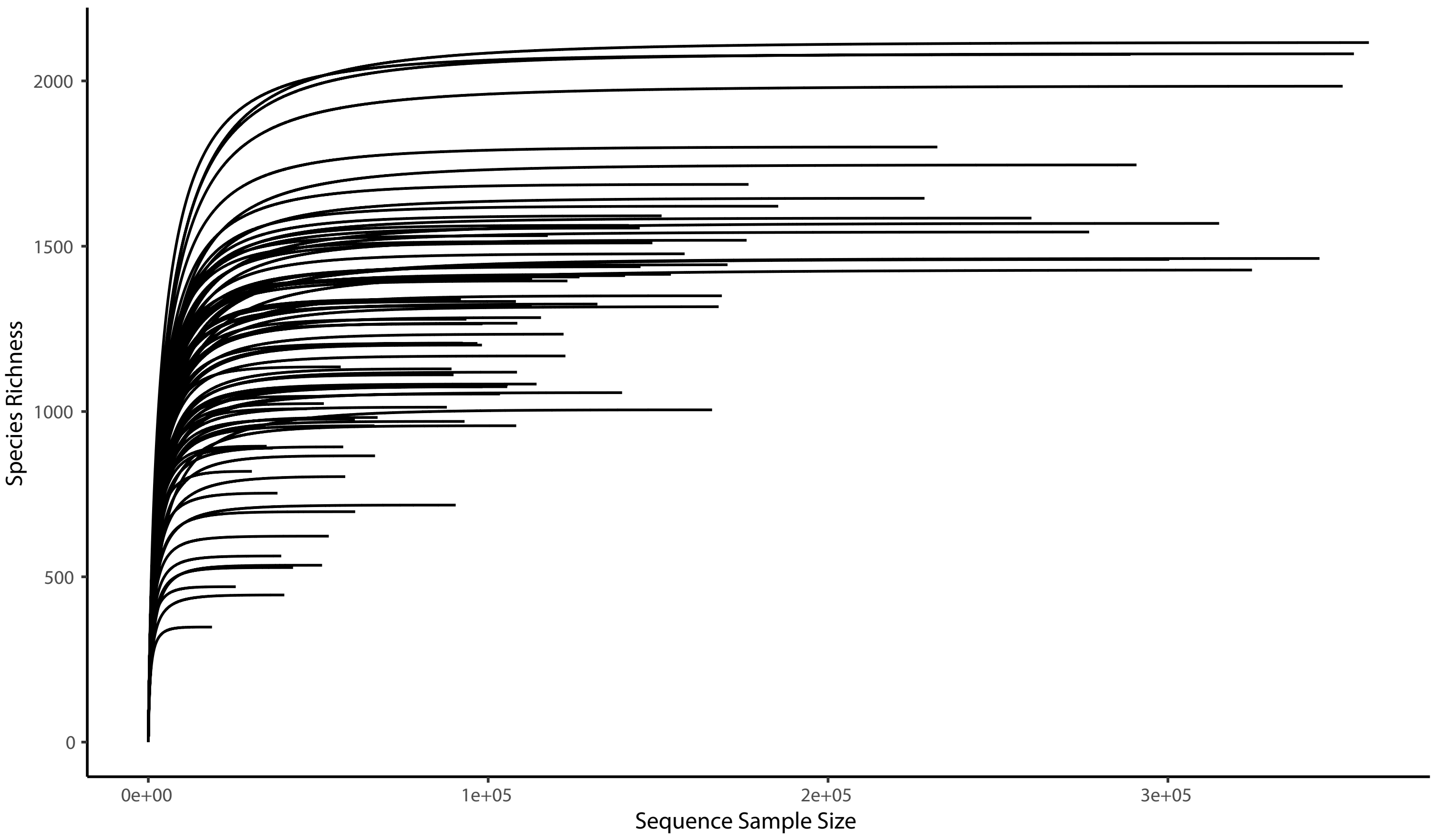

Supplement: Supplementary file 2 — Additional file 2: Rarefaction curves on the number of detected ASVs with the increasing number of reads. [file 42523_2022_205_MOESM2_ESM.pdf]
