# Supplementary figures and images for: Seasonal responses and host uniqueness of gut microbiome of Japanese macaques in lowland Yakushima
Source: Anim Microbiome. 2022 Sep 27;4:54. doi: 10.1186/s42523-022-00205-9 (PMC9513907; doi:10.1186/s42523-022-00205-9)

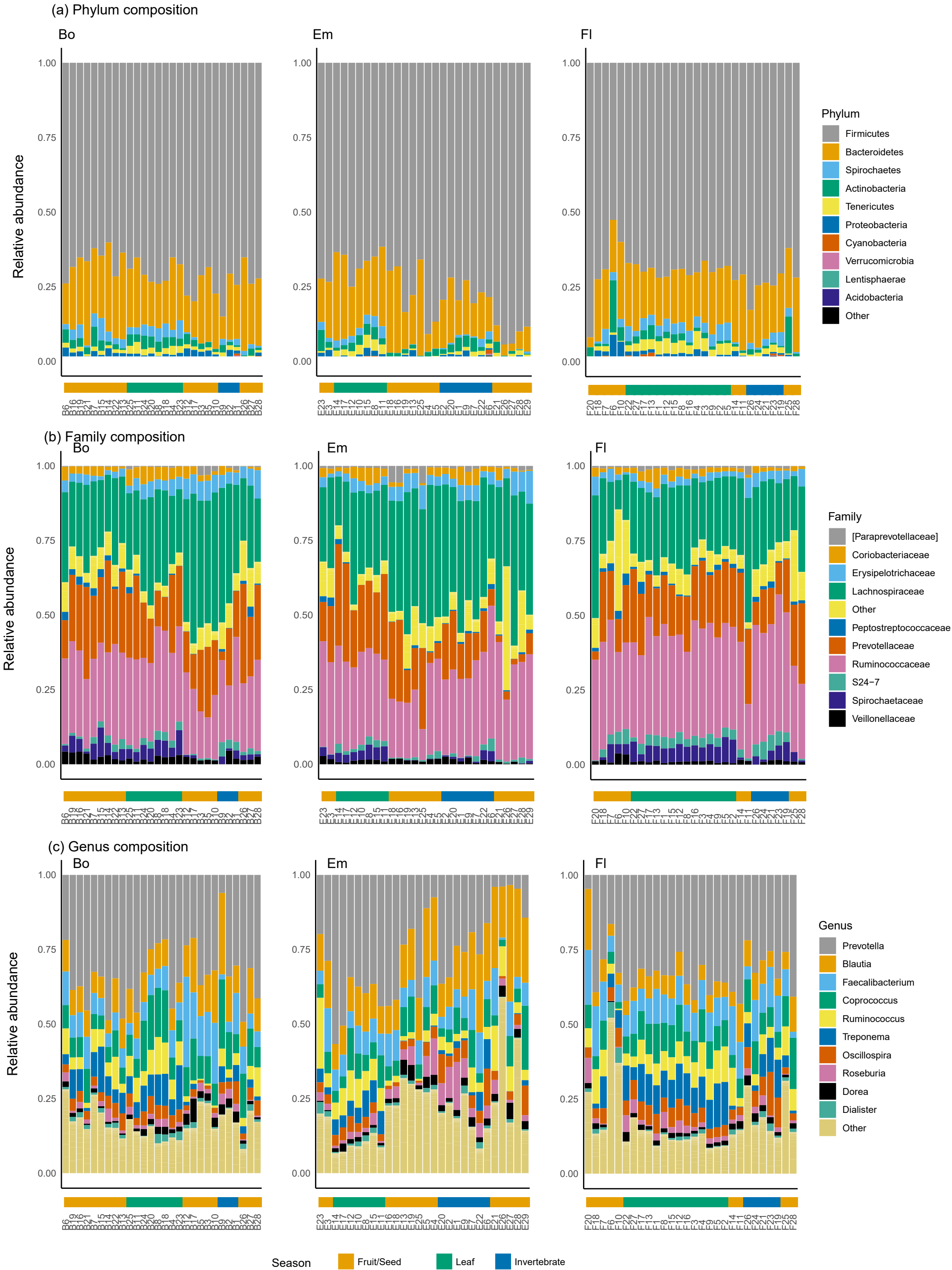

Supplement: Supplementary file 4 — Additional file 4: Phylum, family and genus composition of the gut microbiome of Japanese macaques in the three dietary seasons. [file 42523_2022_205_MOESM4_ESM.pdf]
