# Supplementary material for: Seasonal responses and host uniqueness of gut microbiome of Japanese macaques in lowland Yakushima
Source: Anim Microbiome. 2022 Sep 27;4:54. doi: 10.1186/s42523-022-00205-9 (PMC9513907; doi:10.1186/s42523-022-00205-9)

Supplementary Information 3 Results of the cluster analysis on the monthly variations of dietary composition of the three focal female Japanese macaques

Akiko Sawada, Takashi Hayakawa, Yosuke Kurihara, Wanyi Lee, Goro Hanya

Seasonal responses and host uniqueness of gut microbiome of Japanese macaques in the lowland Yakushima

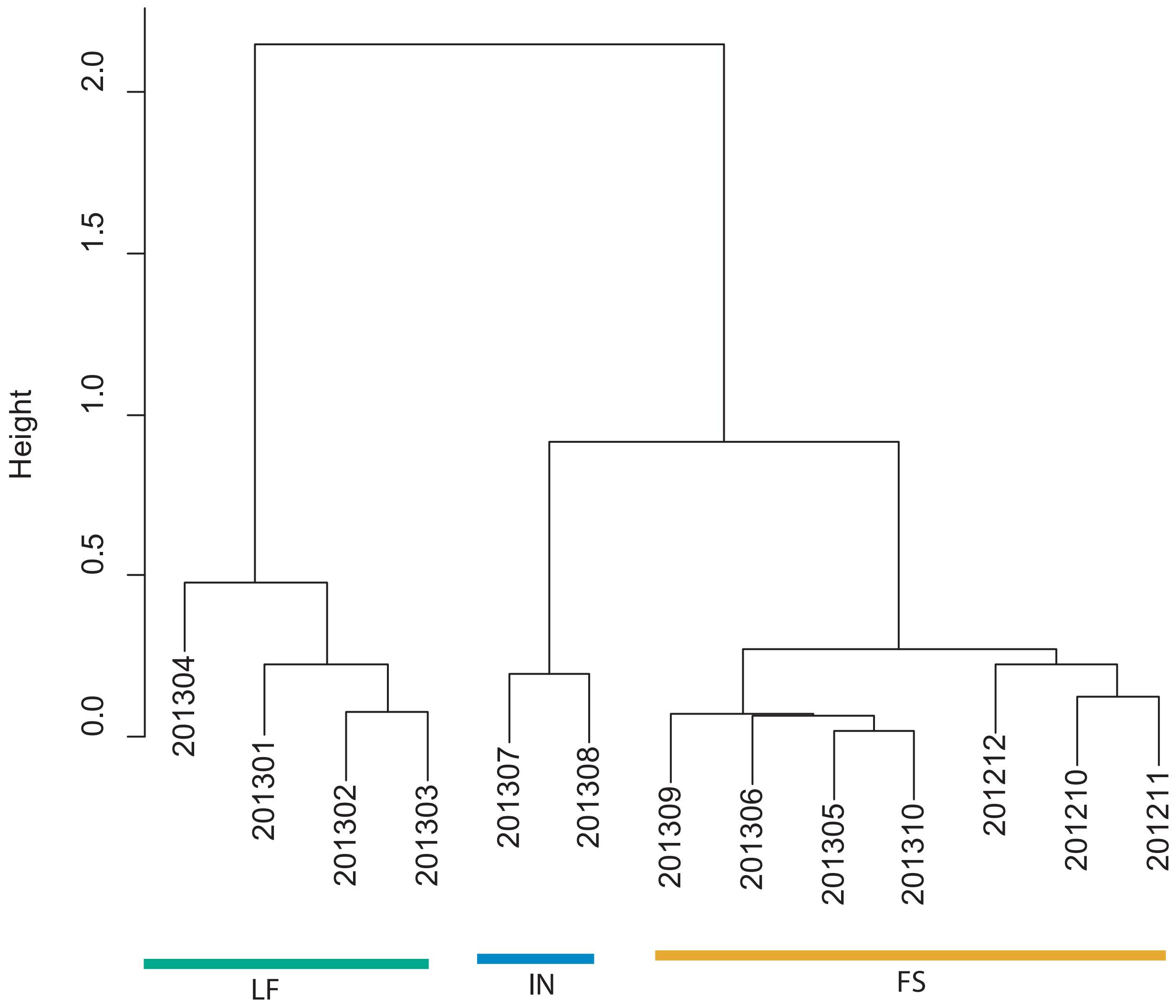

Supplement: Supplementary file 5 — Additional file 5: Results of cluster analysis on the monthly variations of dietary composition of three focal female Japanese macaques. [file 42523_2022_205_MOESM5_ESM.pdf]
